# Analysis of the Variability of the Game Space in High Performance Football: Implementation of the Generalizability Theory

**DOI:** 10.3389/fpsyg.2020.00534

**Published:** 2020-03-25

**Authors:** Rubén Maneiro, Ángel Blanco-Villaseñor, Mario Amatria

**Affiliations:** ^1^Department of Science of Physical Activity and Sport, Pontifical University of Salamanca, Salamanca, Spain; ^2^Faculty of Psychology, University of Barcelona, Barcelona, Spain

**Keywords:** game space, variability, generalizability theory, football, high performance, soccer

## Abstract

The analysis of variability in sport has shown significant growth in recent years. Also, the study of space management in the game field has not been object of research yet. The present study pretends to describe the variability in the use of strategic space in high performance football. To do this, the spatial management of the Spanish men’s soccer team when it is in possession of the ball has been analyzed, during its participation in the UEFA Euro 2012 championship. Specifically, 6861 events have been collected and analyzed. Different zoning of the field have been used, and the location of the ball has been recorded in each offensive action. Using the observational methodology as a methodological filter, two types of analysis have been carried out: first, a General Linear Model was implemented to know the variability of the strategic space. Models with two, three, four and five variables have been tested. In order to estimate the degree of accuracy and generalization of the data obtained, the Generalizability Theory was implemented. Next, and in order to estimate the degree of accuracy and generalization of the data obtained, the Generalizability Theory was implemented. The results showed that the model that produces greater variability and better explanation is the four-variable model (*P* = 0.019; *r*^2^ = 0.838), with the inclusion of the variables match half, rival, move initiation zone and move conclusion zone. Next, an optimization plan was implemented to know the degree of generalization with the Rival, Start Zone (SZ) and Conclusion Zone (CZ) facets. The available results indicate that it is based on an adequate research design in terms of the number of observations. The results of the present study could have a double practical application. On the one hand, the inclusion of the game’s space management in training sessions will potentially conceal the true tactical intention. On the other hand, knowing the variability of the strategic space will allow to exploit areas of the optimal playing field to attack the rival team.

## Introduction

Human behavior is inherently variable. From the evolutionary point of view, each individual is different in terms of genetic inheritance, previous experience and environmental factors (Polderman et al., 2015). These differences determine how each person observes and interacts with the surrounding environment.

Phylogenetically, any human being needs to be able to react adequately to the environmental changes that occur in their habitat. The existence of behavioral variability makes it possible to adapt to these changes (Veá, 1990). Duarte et al. (2012), named as interindividual variability. Interindividual variability is defined as the diversity presented by individuals of the same population in a given situation and at a given time, and which also produces a continuous supply of new solutions to behavioral challenges in front of other groups. On the other hand, Saw et al. (2016), define intra-individual variability as the differentiation of human behavior and responses to the same situation.

Interindividual variability is present in any complex system of nature. In microbiology, an example of adaptation to the environment is that of the amoeba dictyostelium. This amoeba, in a hostile environment with a low amount of nutrients, reproduces adaptive mechanisms of social cooperation and behavioral variability. It goes from an individual state to a socialized state, adapting to the environment and adopting a multicellular structure behavior until the environment becomes favorable again, recovering its individual state (Flowers et al., 2010). This ability to adapt, based on creative behaviors in response to newly encountered problems, will reduce the uncertainty, favoring the individual’s adaptation to the environment in which they live, creating a functional specialization (Wilson and Wilson, 2007).

From the point of view of the ecological dynamics of team sports, the environment in which many sporting events occur is also chaotic and unpredictable. It must be taken into account that each sporting event, each match or episode of confrontation in the area of sports is unique and unrepeatable. There are no two equal matches, nor two identical competitions. Therefore, the flexibilization of motor, technical, tactical and strategic behaviors, concretized in a wide inter and intra-individual variability (Moura et al., 2013; Casal et al., 2016; Maneiro et al., 2019b), will allow the emergence of solutions to changing and unpredictable environmental problems. Adaptation to the environment is essential (Seifert et al., 2016).

Despite its importance, studies on the analysis of variability in sports science seem scarce. Recent works have focused their efforts on measuring heart rate variability in competition (Moreno et al., 2013), in collective sports such as basketball (Bourbousson et al., 2010) or handball (Wagner et al., 2012). As well as in individual sports such as horse riding (Schmidt et al., 2010), cycling (Stanley et al., 2013) and athletics (Cazzola et al., 2016).

Regarding football, most of the works refer again to the variability of heart rate during the competition (Oliveira et al., 2013; Rave et al., 2018). Other works have analyzed the variability of the displacement of the soccer player (Castellano et al., 2011; Couceiro et al., 2014; Castellano and Blanco-Villaseñor, 2015). Other authors have focused their efforts on tactical variability (Okihara et al., 2004; Moura et al., 2013; Maneiro et al., 2017). The work of Frencken et al. (2012), discovered that the greatest physical variability ratios are associated with transcendental moments of the game, such as the goal or shot to goal. On the other hand, the study of Moura et al. (2015), which analyzed positional variability, affirms that the midfielders are the ones with the greatest variability, followed by side players. They conclude their study stating that the finalist teams have more physical variability than the others. On the other hand, the work of Clemente et al. (2019), analyzed the variability in small-sided games in training football and conclude that formats such as 3 × 3 demand higher technical variability ratios than other formats.

Although all these works have a special importance in the applied field, physical and tactical variability is only a small segment of the competitive reality. Soccer is also a sport where space management can be a determining factor, according to reference authors (Grèhaigne, 1992; Garganta, 1997).

The term space was studied in different scientific areas throughout the centuries. From the field of mathematics, Poincaré (1944) said that anyone who speaks of space should never do so in absolute terms, since *it would be a meaningless word*. The author referred to the fluctuating nature of space, its flexibility, its mobile nature.

In Sports Science, the conceptualization of space as part of the internal logic in sport has been collected among other authors by Parlebás (1981) and Hernández-Moreno (1994). Parlebás (1981) distinguishes two types of spatial situations where motor interaction occurs: (1) those in which the formal space is stable and standardized; and (2) those in which the formal space is a carrier of uncertainty. Hernández-Moreno (1994), includes time, regulation, strategy, technique, tactics and playing space as the main parameters that shape the structure of sports.

Some authors who have approached the study of space as a strategic element in high performance football have been; Harris and Reilly (1988), Grèhaigne (1992), Garganta (1997), Castelo (1999), Blanco-Villaseñor et al. (2000), Castellano and Hernández-Mendo (2000), Seabra and Dantas (2006), Castellano et al. (2009), Castellano et al. (2013), Amatria et al. (2019a, b), Gonçalves et al. (2019). Garganta (1997), one of the first authors to speak of *effective playing space*, defines it as *conformational space*, and states that “*it is important to recognize that the concept of space and its ideomotor representation are not limited to the dimensions and physical marks marked on the playing field. The player constructs another self-reference playing space within the physical restrictions imposed by the regulation. This space is the conformational space. It is a configuration or information space, which results from its interaction with the other elements: ball, teammates, opponents, etc., depending on their perception, knowledge and action* (Figures 2, 3)” p. 202.

Mombaerts (1996), affirms that the strategic space conditions and modifies the motor plane, the tactical development and the density of players, since they must “use the resources available in a time and space of a game.” On the other hand, Harris and Reilly (1988), speak of an individual and collective space within the regulatory physical space. More recent studies such as Gonçalves et al. (2019), confirm that teams prioritize some spaces over others based on the quality of the rival.

Taking into account everything said until now, to assess the possible existence of changes in how teams manage the game space, this paper aims to study the variability in the management of strategic space in high performance football. Considering the spatial variables to analyze the game action in football is a relevant proposal to explain the unstable balance of the complex dynamics of the game (Castellano et al., 2013), allowing to increase knowledge about spatial strategic requirements.

For this, the methodological filter will be observational methodology (Anguera, 1979), one of the methodologies that allows the integration of quantitative and qualitative data (Anguera et al., 2014). The application of the observational methodology will allow us to achieve the objectives set out in this work, which are: on the one hand, through a General Linear Model (GLM), we tried to know the variability in the use of space in high performance football; and, on the other hand, through the implementation of the Generalizability Theory (GT) (Blanco-Villaseñor, 1989; Scholotzhaver and Littell, 1997; Blanco-Villaseñor and Anguera, 2000), to estimate the degree of precision and generalization of the results obtained.

This study contributes to increasing the scientific literature on the variability of the game space. The results obtained could be used to assess how the strategic space evolves and, therefore, to have information to propose specific intervention strategies according to the objectives set by football coaches during the season regarding issues such as: planning strategies for training, tactical planning of the team, exploiting possible spatial weaknesses of the rivals or the recognition of potential useful playing spaces.

## Materials and Methods

### Design

We used observational methodology and applied the observational design I/P/M, which stands for Idiographic/Point/Multidimensional. It was idiographic because we studied one team considered as a single unit, point because we studied a single competition (Anguera and Hernández-Mendo, 2013), albeit with an intrasessional follow-up, and multidimensional because we analyzed the multiple dimensions that constituted the *ad hoc* observation instrument used (Anguera et al., 2011). Observation was non participatory and governed by scientific criteria, and the level of perceptibility was complete. To foster methodological quality, an analysis of the different minimum basic elements necessary in Table 1 has been included (Chacón-Moscoso et al., 2018, 2019).

**TABLE 1:** Minimum basic elements.

**Dimension**	**Ítems**
Dimension 1. Extrinsic characteristics	Ítem 1: journal

Dimension 2.	Ítem 2: soccer
Objectives delimitation	Ítem 3: yes
	Ítem 4: yes
	Ítem 5: Yes, with complete empirical definition of constructs and Regulation
	Ítem 6: yes
	Ítem 7: non-participating

Dimension 3. Observational	Ïtem 8: Idiographic/One-time/Multidimensional
design	Ítem 9: yes
	Ítem 10: no

Dimension 4. Participants	Ítem 11: 19 or more
	Ítem 12: no
	Ítem 13: high
	Ítem 14: team sport
	Ítem 15: professionals
	Ítem 16: no
	Ítem 17: no
	Ítem 18: no
	Ítem 19: Worldwide competition

Dimension 5.	Ítem 20: neutral game
Context (setting)	Ítem 21: yes
	Ítem 22: yes
	Ítem 23: yes, between-session constancy
	Ítem 24: 0
	Ítem 25: 0
	Ítem 26: yes
	Ítem 27: no
	Ítem 28: According to activity by play
	Ítem 29: yes
	Ítem 30: yes

Dimension 6. Observational instrument	Ítem 31: Combination of field format and categories system
	Ítem 32: yes
	Ítem 33: yes
	Ítem 34: yes
	Ítem 35: Availability of theoretical framework
	Ítem 36: yes
	Ítem 37: yes

Dimension 7.	Ítem 38: yes
Recording instrument	Ítem 39: Free
	Ítem 40: Direct
	Ítem 41: LINCE
	Ítem 42: EDU-G
	Ítem 43: S.A.S. and EDU-G

Dimension 8. Data	Ítem 44: Type IV data.
	Ítem 45: Sequential data of multi-event.
	Ítem 46: Differentiation of sessions
**Dimension**	**Ítems**

Dimension 9. Parameters specification	Ítem 47: Dynamic behavioral indicators related to sequential structure of the behavior
	Ítem 48: Modified frequency
Dimension 10.	Ítem 49: 42
Observational sampling	Ítem 50: 126
	Ítem 51: 126
	Ítem 52: Mixed
	Ítem 53: no
	Ítem 54: Continuous recording

Dimension 11.	Ítem 55: Linear correlation coefficient
Data quality control	Ítem 56: Quantitative method, duration: Kappa coefficient
	Ítem 57: Global, Pearson correlation
	Ítem 58: Global, Pearson correlation
	Ítem 59: Scores generalizability: to observe the extent to which data do not depend on the person who gets these data

Dimension 12. Data analysis	Ítem 60: Multivariate analysis

### Participants

We used a convenience sample consisting of offensive actions by the Spanish national soccer team during its participation in the UEFA Euro 2012. 6861 multi-events were analyzed. Intersessional consistency throughout the competition was ensured by the fact that all the matches observed were played by the same team (with the same players and shirt numbers) and on the same sized field divided into identical zones. The data has been treated according to the Helsinki declaration.

### Previous General Considerations

To prepare the observation instrument, part of this study relies on different criteria and categories included in previous works. The variables collated as significant in other works have been: Move initiation zone, Move conclusion zone (CZ), Players (Maneiro and Amatria, 2018; Maneiro et al., 2019a); Start sector, Finish sector, Start corridor and Finish corridor (Garganta, 1997; Castelo, 1999); the variables Match half and Phase are aspects included in the institutional regulations of the competition.

### Observation Instrument

**FIGURE 1:**
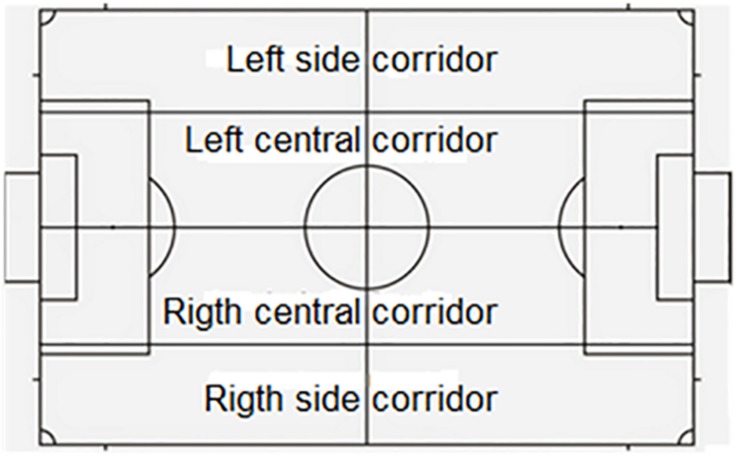
Pitch áreas. Source: Maneiro and Amatria (2018), and Maneiro et al. (2018).

**FIGURE 2:**
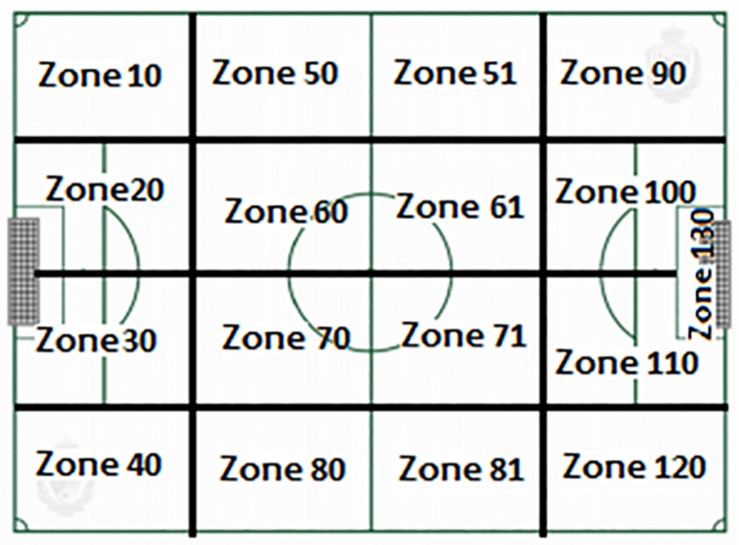
Pitch áreas: sectors. Source: Garganta (1997).

**FIGURE 3:**
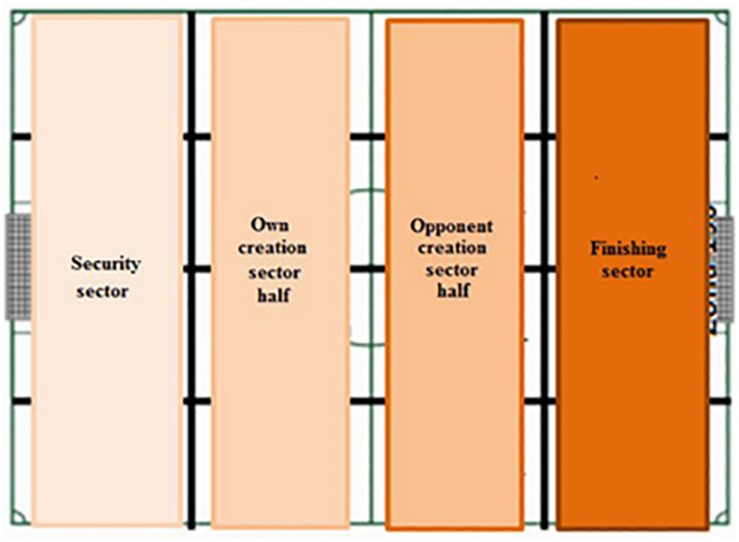
Pitch áreas: corridor. Source: Modified from Garganta (1997).

This instrument (Table 2) is a combination of a field format and category systems (Anguera et al., 2017). The divided field has also been used as shown in Figures 1–3. The category systems are nested within the field format and contain exhaustive, mutually exclusive categories (Anguera and Hernández-Mendo, 2013).

**TABLE 2:** Observation instrument.

**Criteria**	**Category**
1- Rival	1-Italy (groups); 2- Ireland; 3-Croatia; 4-France; 5-Portugal; 6-Italy (final)

2- Phase	1-Groups; 2-Quarterfinals; 3-Semifinals; 4-Final

3- Match half	1-First Half; 2-Second Half; 3-Extension

4- Move initiation zone (IZ)	IZ10; IZ20; IZ30; IZ40; IZ50; IZ51; IZ60; IZ61; IZ70; IZ71; IZ80; IZ81; IZ90; IZ100; IZ110; IZ120

5- Move conclusion zone (CZ)	CZ10; CZ 20; CZ 30; CZ 40; CZ 50; CZ 51; CZ 60; CZ 61; CZ 70; CZ 71; CZ 80; CZ 81; CZ 90; CZ 100; CZ 110; CZ 120

6- Finish	1- No 2- Yes

7- Actions	Numerical

8- Players	Numerical

9- Start sector	1- Security Sector –IZ10, IZ20, IZ30, IZ40-. 2- Own creation sector half –IZ50, IZ60, IZ70, IZ80-. 3- Opponent creation sector half –IZ51, IZ61, IZ71, IZ81-. 4- Finishing Sector –IZ90, IZ100, IZ110, IZ120, IZ130-.

10- Finish sector	1- Security sector –CZ10, CZ20, CZ30, CZ40-. 2- Own creation sector half –CZ50, CZ60, CZ70, CZ80-. 3- Opponent creation sector half –CZ51, CZ61, CZ70, CZ81-. 4- Finishing Sector –CZ90, CZ100, CZ110, CZ120, CZ130-.

11- Start corridor	1- Left side corridor –IZ10, IZ50, IZ51, IZ90-. 2- Left central corridor –IZ20, IZ60, IZ61, IZ100-. 3- Right central corridor –IZ30, IZ70, IZ71, IZ110-. 4- Right side corridor –IZ40, IZ80, IZ81, IZ120-.

12- Finish corridor	1- Left side corridor –CZ10, CZ50, CZ51, CZ90-. 2- Left central corridor –CZ20, CZ60, CZ61, CZ100-. 3- Right central corridor –CZ30, CZ70, CZ71, CZ110-. 4- Right side corridor –CZ40, CZ80, CZ81, CZ120-.

*Source: Modified from Maneiro and Amatria (2018).*

#### Software Tools

The data were recorded using the free software tool Lince (v. 1.2.1; Gabin et al., 2012). The interobserver agreement analysis yielded a kappa value of 0.95 (Table 3).

**TABLE 3:** The interobserver agreement analysis for each criteria.

**Dimension**	**Categories**	**Kappa**	**Agreement**
Rival	1-Italy (groups); 2- Ireland; 3-Croatia; 4-France; 5-Portugal; 6-Italy (final)	1.00	100%

Phase	1-Group; 2-Quarter-finals; 3-Semifinals; 4-Final	1.00	100%

Move initiation zone	IZ10; IZ20; IZ30; IZ40; IZ50; IZ60; IZ70; IZ80; IZ51; IZ61; IZ71; IZ81; IZ90; IZ100; IZ110; IZ120; IZ130	1.00	100%
Move conclusion zone	CZ10; CZ20; CZ30; CZ40; CZI50; CZ60; CZ70; CZ80; CZ51; CZ61; CZ71; CZ81; CZ90; CZ100; CZ110; CZ120; CZ130	0.95	96%

Finish	1-No 2- Yes	1.00	100%

Start sector	1- Security sector –IZ10, IZ20, IZ30, IZ40-. 2- Own creation sector half –IZ50, IZ60, IZ70, IZ80-. 3- Opponent creation sector half –IZ51, IZ61, IZ70, IZ81-. 4- Finishing sector –IZ90, IZ100, IZ110, IZ120, IZ130-.	1.00	100%

Finish Sector	1- Security sector –CZ10, CZ20, CZ30, CZ40-. 2- Own creation sector half –CZ50, CZ60, CZ70, CZ80-. 3- Opponent creation sector half –CZ51, CZ61, CZ70, CZ81- 4- Finishing sector –CZ90, CZ100, CZ110, CZ120, CZ130-.	1.00	100%

Start corridor	1- Left side corridor –IZ10, IZ50, IZ51, IZ90 2- Left central corridor –IZ20, IZ60, IZ61, IZ100-. 3- Right central corridor –IZ30, IZ70, IZ71, IZ110 4- Right side corridor –IZ40, IZ80, IZ81, IZ120-.	1.00	100%

Finish corridor	1- Left side corridor –CZ10, CZ50, CZ51, CZ90-. 2- Left central corridor –CZ20, CZ60, CZ61, CZ100-. 3- Right central corridor –CZ30, CZ70, CZ71, CZ110 4- Right side corridor –CZ40, CZ80, CZ81, CZ120-.	1.00	100%

#### Procedure

The hierarchy of observation units, ranging from molecular to molar, is formed by event (technical actions, play interruptions, and interceptions), sequence of play, and match. A sequence of play, or move, was defined as the series of events that occur from the moment the team being observed gains possession of the ball to the moment it loses it. The sum of sequences constitutes a match. The observation sample for the offensive actions by the Spanish national team during UEFA EURO 2012 contained 6861 events. Type IV data were collected, which means they are concurrent, time-based data.

#### Data Analysis

First, a GLM was applied from SAS for Windows 2010 (SAS Institute Inc., 2004), with the aim of analyzing the variability of the data collected. GLM is a flexible generalization of the ordinary linear regression that includes response variables that have error distribution models other than a normal distribution. The GLM arises from the need to quantitatively express relationships between a set of variables, in which one of them is called a dependent variable, and the remaining are called independent variables.

Next, and with the objective of ensuring that the sample used is reliable, accurate, valid and generalizable, the GT was implemented (Cardinet et al., 2010; Blanco-Villaseñor et al., 2014). The software used to carry out the Generalizability analysis has been SAS (Scholotzhaver and Littell, 1997) and EDU-G (Cardinet et al., 2010). Through the GT it is intended to analyze the different sources of variation that may be affecting the measure, as well as estimate the sampling error of the collected behaviors. The GT allows to search estimates of reliability and margins of error, since it is sufficiently globalizing to adapt to the particular conditions of each measurement object, in this case high performance football.

## Results

Of the total of 12 behavior-criteria, combinations have been made between them to see which of these combinations produce greater variability and better explanation.

Table 4 shows the variability values with only two variables, specifically IZ and CZ. As it can be observed, the values between the start zone and the CZ have statistically significant differences (*P* ≤ 0.0001), although their explanatory capacity is reduced (*r*^2^ = 0.416940). On the other hand, in the random model (Type I SS), where all the variables are chosen randomly, the variables IZ and CZ analyzed in isolation are significant, as well as the interaction IZ^∗^CZ (*P* ≤ 0.0001).

**TABLE 4:** Model with two variables.

**GLM_IZ_CZ**							
**GLM Procedure**							
**Class level information**							
**Class**	**Levels**	**Values**			
Count	7	1 2 3 4 5 6 7			
Pha	4	1 2 3 4			
R	6	1 2 3 4 5 6			
IZ	16	10 100 110 120 20 30 40 50 51 60 61 70 71 80 81 90			
CZ	16	10 100 110 120 20 30 40 50 51 60 61 70 71 80 81 90			
Fin	2	0 1			

Number of read observations 745							
Number of used observations 745							

**Source**	**DF**	**Sum of squares**	**Mean square**	***F*-Value**	**Pr > F**

Model	167	742.239623	4.44454	2.47	<0.0001
Error	577	1037.967088	1.798903	–	–
Total correct	744	1780.206711	–	–	–

**R-square**		**Coef Var**	**MSE Root**	**Count mean**	

**0.416940**		39.63577	1.341232	3.383893	

**Source**	**DF**	**Tipo I SS**	**Mean square**	***F*-Value**	**Pr > F**

IZ	15	92.3365304	6.1557687	3.42	<0.0001
CZ	17	255.8652746	15.0508985	8.37	<0.0001
IZ*CZ	135	394.0378180	2.9187987	1.62	<0.0001

Next, Table 5 shows the results with a model made up with 3 variables, specifically (R, IZ, and CZ). In view of the available results, the model is significant (*P* < 0.001), with an outstanding explanatory capacity (*r*^2^ = 0.728404). On the other hand, the three variables treated independently in the random model are significant by themselves (*r* = 0.0005; IZ = *P* < 0.0001; CZ = *P* < 0.0001), as well as the interaction IZ^∗^CZ (*P* = 0007).

**TABLE 5:** Model with three variables.

**GLM_r_IZ_CZ**							
**GLM Procedure**							
**Class level information**							
**Class**	**Levels**	**Values**			

Count	7	1 2 3 4 5 6 7			
Pha	4	1 2 3 4			
R	6	1 2 3 4 5 6			
IZ	16	10 100 110 120 20 30 40 50 51 60 61 70 71 80 81 90			
CZ	16	10 100 110 120 20 30 40 50 51 60 61 70 71 80 81 90			
Fin	2	0 1			

Number of read observations 745							
Number of used observations 745							
**Source**	**DF**	**Sum of squares**	**Mean square**	***F*-Value**	**Pr > F**

Model	472	1296.709092	2.747265	1.55	<0.0001
Error	272	483.497619	1.777565	–	–
Total correct	744	1780.206711	–	–	–

**R-square**		**Coef Var**	**MSE Root**	**Count mean**	

**0.728404**		39.39999	1.333253	3.383893	

**Source**	**DF**	**Tipo I SS**	**Mean square**	***F*-Value**	**Pr > F**

R	5	40.7036506	8.1407301	4.58	0.0005
IZ	15	91.3061201	6.0870747	3.42	<0.0001
R*IZ	70	153.8869358	2.1983848	1.24	0.1191
CZ	17	218.0828541	12.8284032	7.22	<0.0001
R*CZ	72	121.8964568	1.6930063	0.95	0.5878
IZ*CZ	130	369.3525555	2.8411735	1.60	0.0007
R *IZ*CZ	163	301.4805195	1.8495737	1.04	0.3839

As shown in Table 6, if instead of 3, 4 variables are considered (Pha, R, IZ, and CZ), the results are even better. In this case, the model is again significant (*P* = 0.019), with a high explanatory capacity (*r*^2^ = 0.838). In addition, in the random model, the variables R (*P* = 0.0005), IZ (*P* ≤ 0.0001) and CZ (*P* ≤ 0.0001), are statistically significant by themselves, as well as the interaction IZ^∗^CZ (*P* = 0.0007).

**TABLE 6:** Model with four variables.

**GLM_Pha_r_IZ_CZ**							
**GLM Procedure**							
**Class level information**							
**Class**	**Levels**	**Values**			

Count	7	1 2 3 4 5 6 7			
Pha	4	1 2 3 4			
R	6	1 2 3 4 5 6			
IZ	16	10 100 110 120 20 30 40 50 51 60 61 70 71 80 81 90			
CZ	16	10 100 110 120 20 30 40 50 51 60 61 70 71 80 81 90			

Number of read observations 745					
Number of used observations 745					

**Source**	**DF**	**Sum of squares**	**Mean square**	***F*-Value**	**Pr > F**

Model	593	1492.373378	2.516650	1.32	0.0193
Error	151	287.833333	1.906181	–	–
Total correct	744	1780.206711	–	–	–

**R-square**		**Coef Var**	**MSE Rot**	**Count mean**	

0.838315		40.80050	1.380645	3.383893	

**Source**	**DF**	**Tipo I SS**	**Mean square**	***F*-Value**	**Pr > F**

pha	3	11.6259345	3.8753115	2.03	0.1117
R	5	35.1915127	7.0383025	3.69	0.0035
pha*r	5	27.4120714	5.4824143	2.88	0.0164
IZ	15	88.0734803	5.8715654	3.08	0.0002
pha*IZ	36	80.5466934	2.2374081	1.17	0.2506
R*IZ	69	132.8969170	1.9260423	1.01	0.4697
pha*r*IZ	59	163.9567500	2.7789280	1.46	0.0353
CZ	17	194.3856203	11.4344483	6.00	<0.0001
pha*CZ	34	48.8157768	1.4357581	0.75	0.8324
R*CZ	70	126.1932656	1.8027609	0.95	0.5972
pha* R *CZ	53	102.4955448	1.9338782	1.01	0.4602
IZ*CZ	119	283.7891622	2.3847829	1.25	0.0966
pha*IZ*CZ	65	99.6954530	1.5337762	0.80	0.8389
R *IZ*CZ	43	97.2951962	2.2626790	1.19	0.2249
pha* R *IZ*CZ	0	0.0000000	–	–	–

Finally, if we take into account the inclusion of 5 variables in the model (Table 7), such as Pha, R, IZ, CZ and Fin, and despite showing a high explanatory capacity (*r*^2^ = 0.855), the results are not significant (*P* = 0.0505). In turn, there are 3 variables that present statistical significance in the random model (*r* = 0.004; IZ = 0.0004; CZ ≤ 0.0001). In this case, although some tendency exists in some interactions (Pha^∗^R^∗^IZ, *P* = 0.053), it is not possible to refer statistically significant relationships.

**TABLE 7:** Model with five variables.

**GLM_Pha_r_IZ_CZ_fin**							
**GLM Procedure**							
**Class level information**							
**Class**	**Levels**	**Values**			

Count	7	1 2 3 4 5 6 7			
Pha	4	1 2 3 4			
R	6	1 2 3 4 5 6			
IZ	16	10 100 110 120 20 30 40 50 51 60 61 70 71 80 81 90			
CZ	16	10 100 110 120 20 30 40 50 51 60 61 70 71 80 81 90			

Number of read observations 745					
Number of used observations 745					

**Source**	**DF**	**Sum of squares**	**Mean square**	***F*-Value**	**Pr > F**

Model	613	1522.540045	2.483752	1.26	0.0505
Error	131	257.666667	1.966921	–	–
Total correct	744	1780.206711	–	–	–

**R-square**		**Coef Var**	**MSE Root**	**Count mean**	

**0.855260**		41.44545	1.402470	3.383893	

**Source**	**DF**	**Tipo I SS**	**Mean square**	***F*-Value**	**Pr > F**

Pha	3	11.6259345	3.8753115	1.97	0.1216
R	5	35.1915127	7.0383025	3.58	0.0046
pha*r	5	27.4120714	5.4824143	2.79	0.0200
IZ	15	88.0734803	5.8715654	2.99	0.0004
pha*IZ	36	80.5466934	2.2374081	1.14	0.2950
R*IZ	69	132.8969170	1.9260423	0.98	0.5310
pha*r*IZ	59	163.9567500	2.7789280	1.41	0.0533
CZ	17	194.3856203	11.4344483	5.81	<0.0001
pha*CZ	34	48.8157768	1.4357581	0.73	0.8561
R *CZ	70	126.1932656	1.8027609	0.92	0.6525
pha*r*CZ	53	102.4955448	1.9338782	0.98	0.5162
IZ*CZ	119	283.7891622	2.3847829	1.21	0.1407
pha*IZ*CZ	65	99.6954530	1.5337762	0.78	0.8676
R *IZ*CZ	43	97.2951962	2.2626790	1.15	0.2709
pha*r*IZ*CZ	0	0.0000000	–	–	–
Fin	1	3.8095238	3.8095238	1.94	0.1664
pha*fin	2	7.6452031	3.8226016	1.94	0.1473
R *fin	5	10.1081208	2.0216242	1.03	0.4040
pha*r*fin	4	3.8574421	0.9643605	0.49	0.7429
IZ*fin	7	4.4425793	0.6346542	0.32	0.9426
pha*IZ*fin	1	0.3037975	0.3037975	0.15	0.6950
R *IZ*fin	0	0.0000000	–	–	–
pha*r*IZ*fin	0	0.0000000	–	–	–
CZ*fin	0	0.0000000	–	–	–
pha*CZ*fin	0	0.0000000	–	–	–
R *CZ*fin	0	0.0000000	–	–	–
pha*r*CZ*fin	0	0.0000000	–	–	–
IZ*CZ*fin	0	0.0000000	–	–	–
pha*IZ*CZ*fin	0	0.0000000	–	–	–
R *IZ*CZ*fin	0	0.0000000	–	–	–
pha* R *IZ*CZ*fin	0	0.0000000	–	–	–

Next, the GT has been implemented with the objective of estimating the number of observational criteria analyzed, while trying to reduce the sampling error. First, the design was applied with 12 variables, of which we have selected 3 that present some variability (R, IZ, and CZ). This step is intended to estimate the number of levels or values that these three facets take, in order to generalize these behaviors.

As shown in Table 8, a high error value exists in the RIF facet (which is triple interaction), with a very high residual variability (31%). The IZ^∗^CZ interaction also has a high residual variability, a quarter of the total with 26.5%.

**TABLE 8:** Sample estimation for the evaluation of rivals, move initiation zone and move conclusion zone.

				**Components**				
**Source**	**SS**	**df**	**MS**	**Random**	**Mixed**	**Corrected**	**%**	**SE**
R	41.00	5	8.20	0.02	0.02	0.02	2.3	0.02
IZ	91.00	15	6.07	0.03	0.03	0.03	3.4	0.02
CZ	218.00	17	12.82	0.11	0.11	0.11	13.9	0.04
RI	154.00	75	2.05	0.10	0.10	0.10	13.2	0.02
RC	121.00	85	1.42	0.07	0.07	0.07	9.7	0.01
IC	369.00	255	1.45	0.20	0.20	0.20	26.5	0.02
RIC	301.00	1275	0.24	0.24	0.24	0.24	31.0	0.01

Total	1295.00	1727	–	–	–	–	100%	–

Taking each variable in isolation, the variable R varies 2.3%; IZ varies 3.4%; and CZ varies 13.9%.

Table 9 shows the optimization plan for the R, IZ, and CZ facets, taking the R variable as the instrumentation facet, and IZ and CZ as the differentiation facets (IF/R). The quotient G is presented at this point, as well as the number of observations in terms of cost/benefit for the R facet.

**TABLE 9:** Optimization plan for rivals, move initiation zone and move conclusion zone IC/R.

	**G-study**		**Option 1**		**Option 2**		**Option 3**		**Option 4**		**Option 5**	
	**Lev.**	**Univ.**	**Lev.**	**Univ.**	**Lev.**	**Univ.**	**Lev.**	**Univ.**	**Lev.**	**Univ.**	**Lev.**	**Univ.**
**R**	**6**	**INF**	**6**	**INF**	**10**	**INF**	**12**	**INF**	**15**	**INF**	**20**	**INF**
I	16	INF	16	INF	16	INF	16	INF	16	INF	16	INF
C	16	INF	16	INF	16	INF	16	INF	16	INF	16	INF
Observ.	1727		1727		2880		3456		4320		5760	
Coef_G rel.	0.83		0.83		0.89		0.91		0.92		0.94	
Rounded	0.83		0.83		0.89		0.91		0.92		0.94	
Coef_G abs.	0.82		0.82		0.89		0.90		0.92		0.94	
rounded	0.82		0.82		0.89		0.90		0.92		0.94	
Rel. Err. Var.	0.07		0.07		0.04		0.03		0.03		0.02	
Rel. Std. Err. of M.	0.26		0.26		0.20		0.19		0.17		0.14	
Abs. Err. Var.	0.07		0.07		0.04		0.04		0.03		0.02	
Abs. Std. Err. of M.	0.27		0.27		0.21		0.19		0.17		0.15	

In the absence of an optimization model, the results show a reasonable degree of generalization of 0.83 with the 1727 observations collected. Instead, the optimization plan determined that 3456 observations would be necessary to obtain a generalizability of 0.91, considering 12 rivals.

Table 10 shows the optimization plan taking IZ as the instrumentation facet, and R and R and CZ as differentiation facets (RF/R). The quotient G is presented at this point, as well as the number of observations in terms of cost/benefit for the IZ facet.

**TABLE 10:** Optimization plan for move initiation zone, move conclusion zone and rivals RC/I.

	**G-study**		**Option 1**		**Option 2**		**Option 3**		**Option 4**		**Option 5**	
	**Lev.**	**Univ.**	**Lev.**	**Univ.**	**Lev.**	**Univ.**	**Lev.**	**Univ.**	**Lev.**	**Univ.**	**Lev.**	**Univ.**
R	6	INF	6	INF	6	INF	6	INF	6	INF	6	INF
**I**	**16**	**INF**	**18**	**INF**	**20**	**INF**	**25**	**INF**	**30**	**INF**	**40**	**INF**
C	16	INF	16	INF	16	INF	16	INF	16	INF	16	INF

Observ.	1727		1944		2160		2700		3240		4320	
Coef_G rel.	0.85		0.87		0.88		0.90		0.92		0.94	
rounded	0.85		0.87		0.88		0.90		0.92		0.94	
Coef_G abs.	0.85		0.86		0.87		0.90		0.91		0.93	
rounded	0.85		0.86		0.87		0.90		0.91		0.93	
Rel. Err. Var.	0.03		0.03		0.03		0.02		0.02		0.01	
Rel. Std. Err. of M.	0.18		0.17		0.16		0.15		0.13		0.12	
Abs. Err. Var.	0.04		0.03		0.03		0.02		0.02		0.01	
Abs. Std. Err. of M.	0.19		0.18		0.17		0.15		0.14		0.12	

In the absence of an optimization plan, the results show again a reasonable degree of generalization of 0.85 with the 1727 observations collected. On the other hand, the optimization plan determined that 2700 observations would be necessary to obtain a generalizability of 0.90, considering 25 starting areas.

Finally, Table 11 shows the results for the optimization plan taking CZ as instrumentation facet, and R and IZ as differentiation facets (RI/F). The quotient G is presented at this point, as well as the number of observations in terms of cost/benefit for the CZ facet.

**TABLE 11:** Optimization plan for move conclusion zone, move initiation zone and rivals RI/C.

	**G-study**		**Option 1**		**Option 2**		**Option 3**		**Option 4**		**Option 5**	
	**Lev.**	**Univ.**	**Lev.**	**Univ.**	**Lev.**	**Univ.**	**Lev.**	**Univ.**	**Lev.**	**Univ.**	**Lev.**	**Univ.**
R	6	INF	6	INF	6	INF	6	INF	6	INF	6	INF
I	16	INF	16	INF	16	INF	16	INF	16	INF	16	INF
**C**	**16**	**INF**	**16**	**INF**	**15**	**INF**	**20**	**INF**	**25**	**INF**	**30**	**INF**

Observ.	1727		1727		1440		1920		2400		2880	
Coef_G rel.	0.84		0.84		0.81		0.85		0.88		0.97	
rounded	0.84		0.84		0.81		0.85		0.88		0.97	
Coef_G abs.	0.81		0.81		0.78		0.82		0.85		0.96	
rounded	0.81		0.81		0.78		0.82		0.85		0.96	
Rel. Err. Var.	0.03		0.03		0.03		0.03		0.02		0.00	
Rel. Std. Err. of M.	0.17		0.17		0.18		0.16		0.14		0.07	
Abs. Err. Var.	0.03		0.03		0.04		0.03		0.02		0.01	
Abs. Std. Err. of M.	0.19		0.19		0.20		0.18		0.16		0.07	

In the absence of an optimization plan, the results show again a reasonable degree of generalization of 0.84 with the 1727 observations collected. On the other hand, the optimization plan determined that 2400 observations would be necessary to obtain a generalizability of 0.88, considering 25 starting areas.

## Discussion

The objective of this study was to know the variability in the use of strategic space in high performance football. For this, the spatial management carried out by the Spanish football team during UEFA Euro 2012 was analyzed. Although in previous studies the variability has already been analyzed based on different aspects, this is the first work to study the variability of strategic space in high performance football through systematic observation. For this purpose, two types of analysis were carried out: on the one hand, a multivariate analysis using a General Linear Model, where the spatial variability developed by the team was analyzed. On the other hand, in order to guarantee the reliability and generalization of the sample, the GT was implemented.

The first aspect that can be highlighted from the results obtained is that the Spanish football team presents spatial variability in its game. Four different models have been tested with the inclusion of 2, 3, 4, and 5 variables that had previously been significant. Of these models, the one that presents statistically significant results (*P* = 0.0193), with the best explanatory capacity (*r*^2^ = 0.8383), is the model that considers 4 variables (Table 6).

Analyzing the multivariate model available, it is possible to verify that the Spanish team performs a strategic management of the space when it is in possession of the ball, at different levels: the team varies the starting areas of the offensive actions with the ball compared to the finishing areas. In addition, it also presents variability and versatility depending on its rivals.

As in previous studies (Ric et al., 2017; Gonçalves et al., 2019), limiting or expanding the strategic space will demand different tactical responses from players. An example of this spatial plasticity is the tactical behavior of teams based on the ball (Riera, 1995). When attacking, the strategic space must be wide, causing the appearance of spaces. When defending, the behaviors must be antagonistic. The strategic space should be as little as possible, congesting the attacking team’s useful areas.

This spatial flexibility is not a loss of structural stability, but a sign of adaptability to the environment.

In this sense, it has been proven that physical variability is a significant variable in order to increase the number of goal shots or goals (Frencken et al., 2012). From this work, we also propose to increase the variability of the strategic space as a significant variable to achieve success. This will hide the true tactical intention, mislead, create false leads to the rival team. Theatricalization is a fundamental aspect in sport. As Garganta (1997) mentions, the space of action can not only be seen as a geometric structure, but as a modifiable framework of thought and action, where the functional space of the game is variable. From this work we talk about a functional plasticity.

Finally, in order to guarantee that the sample used is reliable, accurate and generalizable, the GT was developed (Blanco-Villaseñor et al., 2010; Gálvez-Ruiz et al., 2016; Hernández-Mendo et al., 2016). The results available in Tables 9–11 suggest that the work is based on an adequate research design in terms of the number of teams and matches analyzed, with the possibility of reducing the number of observations and having the same generalization accuracy in subsequent analysis.

It is important to note that in the absence of a model for the 3 variables considered (R, IZ and CZ), and with the available observations (*n* = 1727), the G values (generalizability values) are high, all above 0.83. This data is highlighted, since it emphasizes the robustness and solvency of the observations collected in the absence of an optimization plan.

More specifically, to establish a G index of 0.90 (with sufficient quality and to allow generalizing the results), the variables IZ and CZ would have to go from 16 categories to 25. In applied terms, this could be easily implemented, since recent works have corroborated a similar molecularization of the field, without losing practical functionality (Aranda et al., 2019). Other works have proposed a reduced number of zoning (Casal et al., 2017a, b). In economic terms, moving from 16 zones to 25 would increase the generalization of these variables by 0.05, from 0.84 and 0.85, respectively, to 0.90.

As regards the R variable, in the absence of an optimization plan, the G index is moderately acceptable (0.83 with 1727 observations). On the other hand, doubling the number of observations (*n* = 3456) would allow the validity and accuracy of the data to be increased by 0.08, from 0.83 to 0.91. It has been shown that teams perform strategic space management based on the quality of the rival (Gonçalves et al., 2019).

Finally, some of the weaknesses of this study are that they can only be applied to high-level men’s football. It would be interesting to know what space variability is like in high-level women’s football. On the other hand, it would be important to know how space management is carried out in teams that compete in regular championships, with more training time.

## Conclusion

Football is a struggle for space to solve or cause positional imbalances that emerge from the interaction in the game, taking risks in attack by expanding spaces that the opponent must attend or, on the contrary, grouping to close and balance the defense (Castellano et al., 2013). The variable management of this space will allow the implementation of new game tactics, using the strategic space as a tool to achieve the proposed objectives.

The main conclusions of the present work could be summarized in: (1) the Spanish soccer team performs a variable management of the conformational space, both in the area of initiation of the offensive action as in the area where it finishes; (2) the rival team, and the part of the match in which it is located, are aspects that the team takes into account to prioritize some spaces or others in its offensive process; and (3) the results of the GT provide the results presented with sufficient quality and precision to be generalizable results.

## Practical Applications

The practical applications that derive from this study will allow football coaches to know and assess how space management training can be a variable that can help to achieve success in football. The constant professionalization of football, where players have high physical and technical performance, together with high tactical and strategic standards, leave little room for coaches and players to achieve successful routes to achieve victory. Therefore, the inclusion of spatial variability in training can be an alternative to achieve such success.

## Data Availability Statement

The raw data supporting the conclusions of this article will be made available by the authors, without undue reservation, to any qualified researcher. Requests to access the datasets should be directed to the corresponding author.

## Author Contributions

RM developed the project, collected the data, and wrote the manuscript. MA collected the data and critically supervised the manuscript. ÁB-V implemented the methodological aspects and performed the data analysis. All authors have made a substantial, direct and intellectual contributions to the work, and approved it for publication.

## Conflict of Interest

The authors declare that the research was conducted in the absence of any commercial or financial relationships that could be construed as a potential conflict of interest.

The handling Editor DB is currently co-organizing a Research Topic with one of the authors RM, and confirms the absence of any other collaboration.
